# Analysis of Fouling in Hollow Fiber Membrane Distillation Modules for Desalination Brine Reduction

**DOI:** 10.3390/membranes15120371

**Published:** 2025-12-02

**Authors:** Hyeongrak Cho, Seoyeon Lee, Yongjun Choi, Sangho Lee, Seung-Hyun Kim

**Affiliations:** 1 School of Civil and Environmental Engineering, Kookimin University, 77 Jeongneung-ro, Seongbuk-gu, Seoul 02707, Republic of Korea; rhino@kookmin.ac.kr (H.C.); yeon623@kookmin.ac.kr (S.L.); choiyj1041@kookmin.ac.kr (Y.C.); 2 Water Technologies Innovation Institute and Research Advancement (WTIIRA), Saudi Water Authority (SWA), Al-Jubail 31951, Saudi Arabia; 3 School of Civil Engineering, Kyungnam University, Woryeong-dong, Masanhappo-gu, Changwon-si 01767, Gyeongsangnam-do, Republic of Korea; shkim@kyungnam.ac.kr

**Keywords:** membrane distillation, hollow-fiber module, brine scaling, wetting, fouling morphology

## Abstract

Membrane distillation (MD) is a promising technology for reducing the volume of high-salinity brines generated from desalination plants, yet limited knowledge exists regarding its fouling behavior under long-term operation. In this study, fouling was investigated through the autopsy of a hollow fiber MD module operated for 120 days in a direct contact membrane distillation (DCMD) configuration using real desalination brine. Despite stable salt rejection exceeding 99%, a gradual decline in flux and permeability was observed, indicating progressive fouling and partial wetting. Post-operation analyses, including SEM, EDS, ICP-OES, and FT-IR, revealed that the dominant foulants were inorganic scales, particularly calcium carbonate (CaCO_3_), with minor contributions from suspended particles (SiO_2_, Fe) and organic matter. Fouling was more severe in the inlet and inner regions of the module due to intensified temperature and concentration polarization, which promoted supersaturation and scale deposition. These combined effects led to a reduction in membrane hydrophobicity and liquid entry pressure, ultimately accelerating partial wetting and performance deterioration. The findings provide valuable insights into the spatial fouling behavior and mechanisms in MD systems, highlighting the importance of hydrodynamic optimization and fouling mitigation strategies for long-term brine concentration applications.

## 1. Introduction

With the rapid global expansion of seawater desalination, the sustainable management of brine has emerged as a critical environmental challenge [1]. Brine produced from desalination plants typically exhibits salt concentrations significantly higher than those of the source seawater [2]. In many cases, this brine is discharged directly into the marine environment without prior treatment [3,4]. However, the direct discharge of untreated brine from desalination plants is a growing environmental concern [5,6,7]. Elevated salinity can alter seawater quality, influencing both abiotic (chemical and physical) conditions and biotic (living organisms) components of marine ecosystems [5]. Brine discharge has been shown to degrade habitats, including seagrass meadows and benthic environments, which are vital for maintaining biodiversity and ecosystem services [8]. In addition, brine often contains residual process chemicals—such as antiscalants, antifoulants, and anticorrosion agents—that may further compromise marine life and ecosystem health [9,10]. These impacts are particularly severe in environmentally sensitive or stressed regions, including arid zones and semi-enclosed seas where water circulation is limited [11].

Membrane distillation (MD) has recently attracted considerable attention as a promising technology for the treatment of desalination brine [12]. As a thermal separation process, MD is not limited by osmotic pressure, enabling the treatment of highly concentrated brines and facilitating near-zero liquid discharge (ZLD) [13,14,15,16,17]. MD operates at low pressures and moderate temperatures, which simplifies installation and operation [18,19]. Moreover, MD can effectively utilize low-grade or waste heat sources, which enhances its sustainability compared to conventional evaporation [20,21]. For instance, photothermal membrane distillation (PMD), which uses light-absorbing layers to reduce bulk heating demand, has demonstrated higher thermal efficiency and the potential for low-grade-heat-driven operation [22]. This advantage makes MD a more attractive option than other thermal processes for brine management [23,24].

In this context, MD is being extensively investigated and applied for desalination brine management, with a special focus on enhancing water recovery, enabling resource recovery, and improving overall system efficiency through process integration [25,26]. Beyond water production, MD has demonstrated the capability to further concentrate brine, thereby allowing resource recovery, such as salt extraction via crystallization [27,28]. Recent studies highlight a trend toward integrating MD with conventional desalination and thermal processes, particularly in cases where low-grade or excess heat is available [26,29]. Furthermore, the performance of various MD configurations, including direct contact MD (DCMD) [30,31], vacuum MD (VMD) [32], and submerged MD (SMD) [33], has been evaluated for the treatment of brine.

Among them, DCMD is the simplest configuration that places the hot feed and the cold product water on opposite sides of a hydrophobic membrane [34]. The temperature difference imposed across the membrane creates a vapor-pressure gradient that drives vapor through the pores to condense in the cold stream [34]. Compared with AGMD (Airgap MD)/VMD, DCMD typically offers higher flux, but results in greater conductive heat loss and stronger temperature polarization [35]. Due to its simplicity, the DCMD configuration has been used in preliminary feasibility studies for brine concentration, waste heat recovery, and compact small-to-medium desalination systems [30,31].

Membrane distillation performance is governed by three coupled phenomena: fouling, scaling, and wetting [36]. Particulate/organic fouling forms conditioning layers that raise mass-transfer resistance [37]; inorganic scaling arises from interfacial supersaturation and boundary-layer effects [37,38]; and wetting occurs when deposits reduce hydrophobicity or alter pore geometry, lowering liquid entry pressure (LEP) [38]. Together, these processes depress flux, destabilize salt rejection, and diminish the effective driving force [39]. Mitigation combines pretreatment/antiscalants, hydrodynamic optimization, operation within LEP safety margins, and periodic, chemistry-matched cleaning, supported by real-time monitoring of key variables such as flux and conductivity [40].

Moreover, the limited experience with long-term operation remains a major barrier to the practical implementation of MD for brine concentration [41,42]. When applied to high-salinity feedwaters such as desalination brine, MD is highly susceptible to fouling, scaling, and wetting [43,44]. Nevertheless, most previous studies have primarily addressed these issues at the laboratory scale under short-term operating conditions [38,45]. While the information from such studies is valuable, they cannot be directly extrapolated to the design and optimization of pilot- or full-scale systems [46,47]. Scaling up from laboratory to pilot plant introduces additional technical complexities, including non-linear performance behaviors and variations in system design and operating characteristics that must be carefully addressed [47,48].

A comprehensive understanding of membrane fouling and scaling during long-term operation is essential for the reliable application of MD [49,50]. Several approaches have been developed to investigate fouling and scaling phenomena, ranging from in situ monitoring techniques [51,52,53] to post-operation analyses [54]. Among these, membrane autopsy is considered the most conclusive method, as it enables direct elucidation of fouling and scaling mechanisms after operation [55,56]. This approach typically includes the disintegration of membrane modules, followed by detailed physical and chemical characterization of the membrane surface and deposits [57]. Membrane autopsy has been widely applied in other pressure-driven membrane processes such as reverse osmosis (RO), providing critical insights into fouling and scaling behaviors [58,59]. However, only limited studies have reported the application of membrane autopsy for MD. A previous study focusing on the long-term performance of MD performed the analysis of membranes after the end of the operation, but did not provide spatially resolved autopsy or quantitative assessment of fouling distribution within hollow-fiber modules [47]. The membrane area was too small (0.0889 m^2^) and thus no spatial distribution of fouling could be identified. Another study summarized various fouling phenomena and characterization methods, including post-operation membrane autopsy, for flat-sheet and laboratory-scale modules under synthetic feed conditions [45]. But it did not involve an in-depth experimental dissection of long-term operated MD modules treating real desalination brine. This highlights the need for further systematic investigation [60].

This study investigates the long-term performance of an MD pilot plant operated with real brine from a seawater desalination facility. The work specifically focuses on characterizing fouling behavior and wetting phenomena during continuous operation, followed by a detailed post-operation autopsy of the MD module. A range of physical and chemical analytical techniques was employed to systematically elucidate fouling mechanisms and pathways. To the best of the authors’ knowledge, this study is the first to present a comprehensive post-operation analysis of a pilot-scale MD module treating real desalination brine, thereby providing critical insights for the future design and scale-up of MD systems.

## 2. Materials and Methods

### 2.1. Feed Water (Desalination Brine)

Table 1 summarizes the water quality parameters of the desalination brine used as feed for the MD pilot plant. The brine, which was supplied from an operating multi-effect distillation (MED) facility in Jubail, Saudi Arabia, exhibited a total dissolved solids (TDSs) concentration of approximately 65,520 mg/L, indicating a high salinity typical of desalination brine. For comparison, the TDS of the seawater was 46,800 mg/L. Sodium (Na^+^) and chloride (Cl^−^) were the predominant ions, while divalent cations such as calcium (Ca^2+^) and magnesium (Mg^2+^) accounted for a total hardness of 10,654 mg/L as CaCO_3_. Sulfate (SO_4_^2−^) and bicarbonate (HCO_3_^−^) were also present at measurable levels, which may contribute to scaling tendencies during concentration. The total suspended solids (TSSs) concentration was 26.6 mg/L, suggesting that the feed contained substantial amounts of particulates. No further pretreatment was carried out for the feed because the aim of this test was to evaluate the fouling and scaling propensity.

### 2.2. MD Module

**Figure 1:**
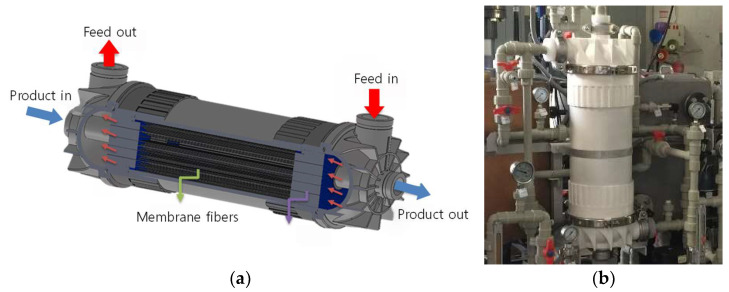
MD membrane module: (**a**) 3D schematics; small red arrows indicate the flow direction of the hot feed along the shell side, and the purple arrow highlights the shell-side feed channel surrounding the membrane fibers. (**b**) photography.

Table 2 summarizes the specifications of the hollow fiber membrane module used in the MD pilot plant. The membrane fibers were fabricated from polyvinylidene fluoride (PVDF), which has excellent chemical resistance and thermal stability. The module was operated in an outside-in flow configuration under a counter-current arrangement between the feed and product water streams. The fibers had an inner diameter of 1.2 mm, an outer diameter of 0.7 mm, and an average wall thickness of 0.25 mm. The effective length of the fiber was 400 mm. The membrane exhibited a tensile strength of 9.05 MPa and an elongation ratio of 70%. PVDF hollow-fiber tensile strengths are reported in the 2–6 MPa range depending on formulation/process [61], indicating our measured 9.05 MPa is above typical PVDF membrane values. The effective membrane area of the module was 2.3 m^2^, comparable to reported values of pilot-scale MD modules ranging from 0.66 m^2^ to 7 m^2^ [62]. A schematic representation of the module design and its actual installation in the pilot system are shown in Figure 1a and Figure 1b, respectively.

### 2.3. MD Pilot Plant

**Figure 2:**
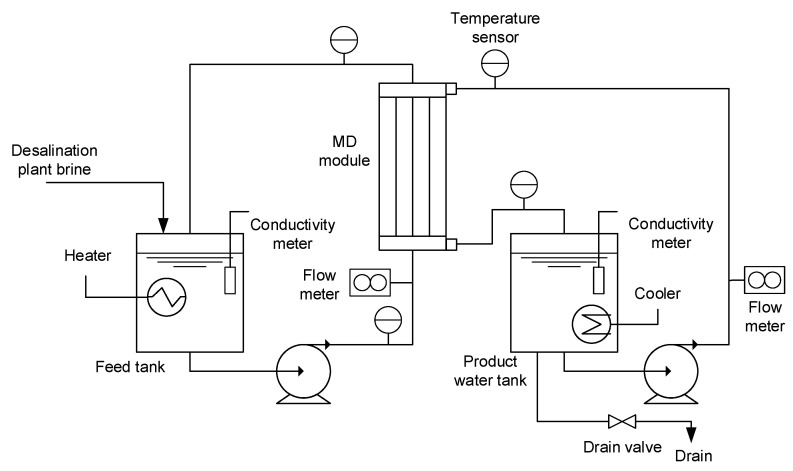
Schematics of the MD pilot plant.

The schematic diagram of the MD pilot plant is presented in Figure 2. The system consisted of a feed tank equipped with an electric heater to maintain the desired temperature and a product water tank equipped with a cooling unit to maintain the temperature difference required for MD operation. Conductivity and temperature sensors were installed on both the feed and product water sides. Flow meters were placed along the feed and product water lines to monitor system performance. ΔP was controlled by regulating the pump power, while ΔT was controlled by using the heater in the feed tank and the cooler in the product water tank. The flux and conductivity were measured every 10 min, and the associated measurement uncertainty was less than 10%, according to the manufacturer’s specifications.

The operating conditions of the MD pilot plant are summarized in Table 3. The pilot plant operated in a direct contact membrane distillation (DCMD) mode, where the feed and permeate streams directly contacted opposite sides of the hydrophobic membrane. Although the feed temperature was set to 60 °C, it varied slightly from 55 to 60 °C due to the changes in the brine temperature and the practical limits of the temperature regulation system in the MD pilot. The feed flow rate was 60 L/min, and the product water flow rate was 40 L/min. The corresponding Reynolds numbers for the feed and product water sides were 10,820 (turbulent) and 20.8 (laminar), respectively. They were computed using the hydraulic diameter and the measured viscosity at a given temperature.

The design flux was 2.0 kg/m^2^-h, and the MD system had a nominal treatment capacity of 0.1 m^3^/day for this given flux value. According to the literature, flat sheet membranes consistently demonstrate higher fluxes (20–30 L/m^2^-h) compared to hollow fiber modules (1–4 L/m^2^-h) under similar temperature conditions [63]. The difference is attributed to factors such as higher distillate flow rates, less confinement of the distillate stream, and potentially better heat management in flat sheet configurations [63]. The recovery of the product water ranged from 0.45 to 0.62. During the whole operation period, no chemical cleaning as well as rinsing/flushing was performed to strengthen the attribution of performance drift to fouling/scaling rather than cleaning artifacts.

### 2.4. Membrane Autopsy

**Figure 3:**
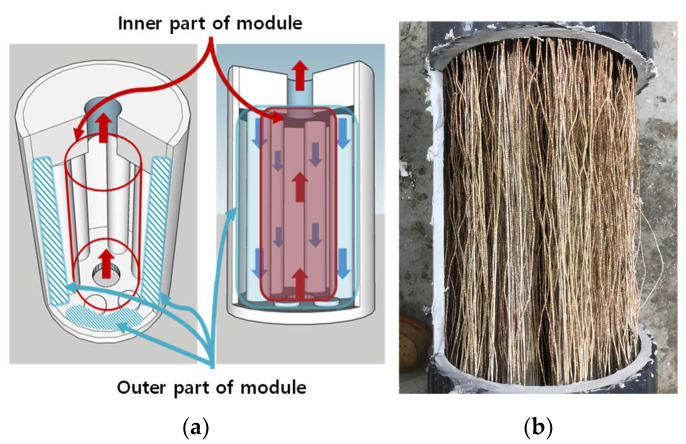
(**a**) Inner and outer parts of the MD module; red regions and arrows denote the hot feed channel and its flow direction, while blue regions and arrows denote the cold permeate/coolant channel flowing in counter-current. (**b**) Disassembly of the MD module for autopsy.

Figure 3 illustrates the internal structure of the membrane distillation (MD) module and its disassembly procedure for autopsy. As shown in Figure 3a, the module was designed with a counter-current flow configuration. After the pilot operation, the module was carefully disassembled, as shown in Figure 3b, to facilitate visual inspection and collection of membrane samples.

Excluding the potting regions, a 34 cm active section of the MD module was divided into two 10 cm segments for membrane sampling and subsequent analysis. The extracted membrane fibers were classified into four groups based on their positions within the module: (1) feed inlet/inner part, (2) feed outlet/inner part, (3) feed inlet/outer part, and (4) feed outlet/outer part. Both physical and chemical analyses were performed on the extracted fibers. Physical characterization included visual inspection and scanning electron microscopy (SEM) to examine the surface morphology and fouling distribution. For chemical analysis, membrane samples (10 cm × 60 fibers each) were immersed in 1 L of ultrapure water and subjected to ultrasonic cleaning (CPX5800H-E, Emerson, Saint louis, MO, USA) for 24 h to detach foulants from the membrane surface for subsequent compositional analysis. It should be noted that ultrasonic cleaning cannot transfer 100% of foulants from membranes [64]. For instance, inefficiency in removing the gel layer and pore-blocking fouling has been reported [65]. Accordingly, the results of the chemical analysis in this study are interpreted as relative values representing overall trends. Details on the analytical methods are provided in the Section 2.5.

### 2.5. Analytic Methods

The extracted hollow fibers were characterized using a combination of morphological, elemental, and chemical analyses. Surface morphology and elemental composition were examined by Scanning Electron Microscopy combined with Energy Dispersive X-ray Spectroscopy (SEM-EDX, SU8700, Hitachi, Tokyo, Japan). The concentrations of inorganic ions in the foulant extracts were measured using ICP-OES (5110, Agilent, Santa Clara, CA, USA). TOC was measured using a TOC analyzer (Multi N/C 3300, Analytic-Jena GmbH+Co. KG, Jena, Germany). Fourier transform infrared (FT-IR) analysis was conducted using a Nicolet iS5 spectrometer (Thermo Fisher Scientific, Waltham, MA, USA). The contact angle (CA) of the membranes was measured by the technique of sessile drop contact angle using a CA measurement device (Smart Drop, Femtobiomed, Seongnam-si, Korea). In addition, the liquid entry pressure (LEP) of the membranes was measured using an in-house LEP apparatus [66,67].

## 3. Results and Discussion

Subsequent sections report long-term MD operation (flux, salt rejection, and permeability), followed by post-operation autopsy of key membrane properties (liquid entry pressure, contact angle, tensile strength), and conclude with visual, morphological, and compositional analyses that elucidate fouling distributions and mechanisms across module regions.

### 3.1. Performance of the MD Module During the Long-Term Operation

#### 3.1.1. Temporal Variations in Flux and Rejection

**Figure 4:**
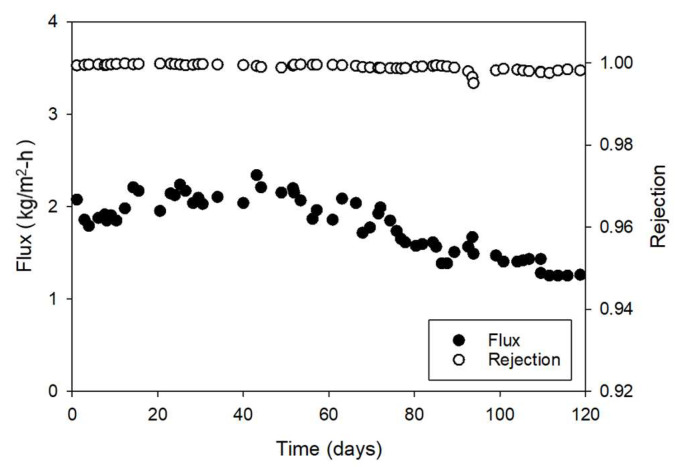
Time-dependent variation in flux (●) and rejection (○) in a hollow fiber MD module during 120 days of desalination brine treatment.

Figure 4 shows the time-dependent variations in flux and rejection of the hollow fiber MD module during 120 days of desalination brine treatment. The initial flux was approximately 2.0–2.23 kg/m^2^·h and gradually decreased over the operation period, reaching about 1.25 kg/m^2^·h after 120 days. This decline can be attributed to progressive membrane fouling and partial wetting that developed over time. It should be noted that no chemical cleaning was carried out during the entire operation period. Accordingly, the flux decline due to fouling and scaling was severe. Under real industrial conditions, periodic flushing and cleaning can slow the progression of fouling and scaling, allowing for longer operation with more stable performance.

The apparent rejection (*R*) by the MD membrane was calculated by:

(1) $$R=1-\frac{c_{p}}{c_{f}}=1-\frac{S_{p}}{100}$$

where *c_p_* is the product water concentration (mg/L), *c_f_* is the feed concentration (mg/L), and *S_p_* is the salt passage. Despite the gradual decline in flux, the salt rejection remained consistently high (>99%) throughout the operation. Minor fluctuations were observed, with rejection values ranging from 0.9951 to 0.9999, corresponding to salt passages of 0.0049 and 0.0001, respectively. The slight increase in salt passage over time indicates the onset of partial pore wetting, which likely occurred concurrently with fouling.

#### 3.1.2. Comparison of Flux and Product Water TDS

**Figure 5:**
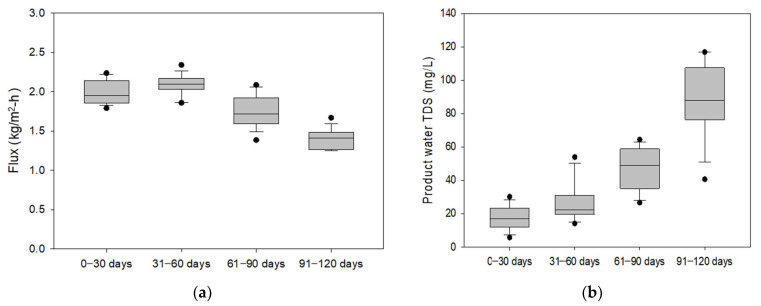
Box plots of (**a**) flux and (**b**) product water TDS over different operational periods (0–30, 31–60, 61–90, and 91–120 days) in a hollow fiber MD module treating desalination brine.

As shown in Figure 5, to further evaluate the changes in the MD performance with time, flux and product water quality were compared over four operational periods. The average flux gradually decreased from approximately 2.1 kg/m^2^·h in the initial stage (0–30 days) to about 1.4 kg/m^2^·h after 120 days, corresponding to a 33% reduction in the flux.

These results indicate the progressive development of fouling after 60 days of operation. In contrast, the product water TDS increased from less than 20 mg/L in the early stage to more than 100 mg/L in the final period (91–120 days), reflecting a gradual loss of hydrophobicity and an increase in salt passage from the beginning. These simultaneous trends in declining flux and rising TDS suggest that both fouling and pore wetting were major contributors to the deterioration of MD performance during long-term operation.

#### 3.1.3. Analysis of Water Permeability and Salt Passage

**Figure 6:**
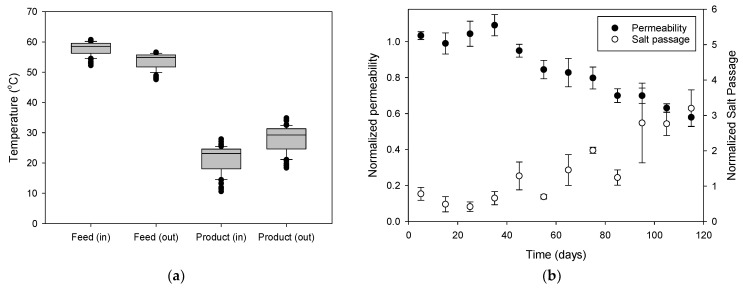
(**a**) Feed and product water temperatures at module inlets and outlets, and (**b**) variation in normalized permeability and salt passage over 120 days of MD operation.

The reported flux decline (~33%) may be attributed to fouling, but part of this decrease could be due to a reduction in ΔT over time. During the MD operation, the temperatures of the feed and product water slightly varied as shown in Figure 6a. The average and standard deviation of the feed temperatures entering the MD module are 57.84 °C and 2.22 °C, respectively. The respective values for the temperatures of the feed leaving the MD module are 53.89 °C and 2.53 °C. The symbols on the ends of the box-and-whisker plots represent outliers, or extreme data points. The temperature difference between the feed and product streams was maintained at approximately 35–40 °C, as indicated by the inlet and outlet temperature distributions.

As variations in temperature affect flux in MD, the two effects should be distinguished. Accordingly, water permeability (*A_m_*) was calculated by dividing MD flux by vapor pressure [68]:

(2) $$A_{m}=\frac{J_{v}}{P_{v}}$$

where *J_v_* is the flux (kg/m^2^-h) and *P_v_* is the vapor pressure (kPa). The following equation was used to calculate *P_v_* as a function of the temperatures [69]:

(3) $$\log P_{v}(T,S)=\left(7.19621-\frac{1730.63}{233.426+T}\right)-\left(2.1609\times 10^{-4}S+3.5012\times 10^{-7}S^{2}\right)$$

where *T* is the temperature (°C) and *S* is the feed salinity (g/L). Then, the normalized permeability (*A_n_*) was calculated at different time (*t*) using the following equation:

(4) $$A_{n}=\frac{A_{m}(t)}{A_{m}(0)}$$

Unlike flux, *A_n_* is unaffected by temperature changes, making it ideal for examining the effects of fouling. The normalized salt passage (*Sp_n_*) was also calculated by:

(5) $$Sp_{n}=\frac{Sp(t)}{Sp(0)}$$

Although *Sp_n_* has not been widely adopted in the literature, it was introduced to conveniently monitor the relative change in salt rejection capability compared with the initial operation stage.

Figure 6b illustrates the temporal variations in normalized permeability and normalized salt passage during the operation. The normalized permeability gradually decreased to approximately 60% of its initial value, indicating progressive fouling and possible wetting of the membrane surface. The normalized salt passage exhibited a gradual increase, suggesting that localized pore wetting occurred as the fouling layer altered the membrane’s hydrophobicity. Notably, a pronounced rise in salt passage (increased to 320% of its initial value) after approximately 95 days implies an acceleration of wetting phenomena at this stage. The simultaneous decline in permeability and increase in salt passage clearly demonstrate the deterioration of membrane performance during long-term operation.

### 3.2. Changes in Physical Properties of the Membrane After Long-Term Operation

#### 3.2.1. Visual Observation of Foul Distribution Within the Module

**Figure 7:**
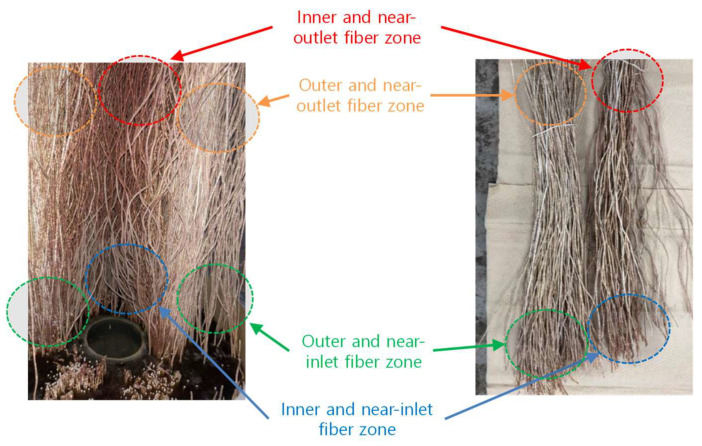
Photographs of hollow fiber bundles after operation, highlighting different zones (inner/outer and near inlet/outlet) for fouling comparison.

Figure 7 shows photographs of the hollow fibers in the MD module after the operation. Different zones within the module were highlighted for comparison. Distinct differences in surface appearance were observed among the inner and outer fibers as well as between the inlet and outlet regions. The fibers located near the inlet exhibited slightly thicker and darker deposits than those near the outlet, suggesting greater fouling intensity near the inlet zone. In particular, the inner fibers showed more severe discoloration and fouling accumulation compared with the outer fibers, indicating that flow channeling and localized temperature gradients may have enhanced deposition in the inner zones [70]. The accumulation of natural organic matter (NOM), humic acids, and other rejected substances on the membrane surface during the MD process can also result in a brown color [71]. Minor co-deposition of iron oxides and hydroxides may also change the membrane to brown [72]. These visual observations provide preliminary evidence of the spatial distribution of foulant deposits within the module, which was further examined through detailed microscopic and chemical analyses.

#### 3.2.2. Comparison of Contact Angles and Liquid Entry Pressure

The contact angle represents the wettability of a membrane surface, defined as the angle formed between a liquid droplet and the membrane surface [73]. Changes in contact angle may suggest either the deposition of hydrophilic foulants or the modified hydrophobic properties of the membrane surface [74]. Table 4 summarizes the contact angles measured for membrane fibers collected from different positions within the MD module. The intact membrane fiber showed a contact angle of 104.65°, which is a typical value for PVDF MD membranes. In contrast, all fouled fibers showed a significant reduction in contact angle, indicating a loss of surface hydrophobicity due to fouling and wetting during the operation. The lowest contact angle (41.26°) was observed in the inner and near-outlet fibers, suggesting that these regions experienced the most severe wetting. This hydrophobicity loss can be attributed to the adsorption of inorganic and organic foulants [75], which induced water penetration into the membrane pores. The spatial variation in contact angle corresponds well with the observed fouling distribution, confirming that wetting was more pronounced along the inner flow path and toward the outlet of the MD module.

The liquid entry pressure (LEP) represents the minimum pressure required for liquid to penetrate through the membrane pores, implying the resistance of the membrane to wetting [76]. As shown in Table 5, the intact membrane fiber exhibited an LEP of 2.78 ± 0.67 bar, which is higher than the proposed value of 2.5 bar to ensure efficient and stable operation [77]. In contrast, the LEP values of the fouled fibers were significantly lower, indicating that fouling and surface wetting during operation reduced the membrane’s resistance to liquid intrusion. The lowest LEP values were observed for the inner and near-inlet (1.21 ± 0.86 bar) and inner and near-outlet (1.18 ± 0.40 bar) fibers, suggesting that these regions experienced the most severe wetting. This decrease in LEP corresponds well with the reduced contact angles observed in Table 4. As reported in the literature, LEP is closely related to the contact angle, as it is governed by the capillary pressure at the liquid-solid interface. As reported in the literature, when the contact angle decreases, the membrane becomes less hydrophobic, leading to a lower LEP and increased likelihood of pore wetting [78]. Accordingly, this confirms that surface hydrophobicity loss and partial pore wetting occurred preferentially in the inner zones of the MD module.

#### 3.2.3. Comparison of Tensile Strength of Membrane Fibers

Tensile strength represents the maximum stress that a membrane fiber can withstand before mechanical failure, reflecting its structural integrity and resistance to deformation [79]. As summarized in Table 6, the intact membrane fiber exhibited a tensile strength of 9.05 MPa. In contrast, all fouled fibers showed reduced tensile strength values, ranging from 5.93 to 6.53 MPa, suggesting that prolonged operation and fouling exposure led to a partial loss of mechanical integrity.

Since no chemical cleaning was carried out during the operation, the decrease in tensile strength can be attributed to fouling-induced stress, localized scaling, and possible microstructural damage within the polymer matrix caused by long-term thermal and hydraulic stresses [45,80]. Among the tested samples, the lowest tensile strength was observed in the inner and near-inlet fibers, implying that these regions were subjected to greater mechanical stress and fouling accumulation during operation. These results indicate that fouling not only affects transport performance but may also compromise the long-term mechanical stability of the MD module. Similar results were also reported in the literature: In a study using hollow fiber membranes for DCMD, it was found that complex produced water (PW) feed had a more adverse effect on the tensile strength of the membrane than saltwater [81]. It was also reported that fouling and scaling may cause membrane partial wetting or severe membrane damage, which can be associated with a loss of mechanical integrity, including tensile strength [82].

### 3.3. Morphological and Compositional Analysis of Fouled Membranes

#### 3.3.1. SEM Analysis

**Figure 8:**
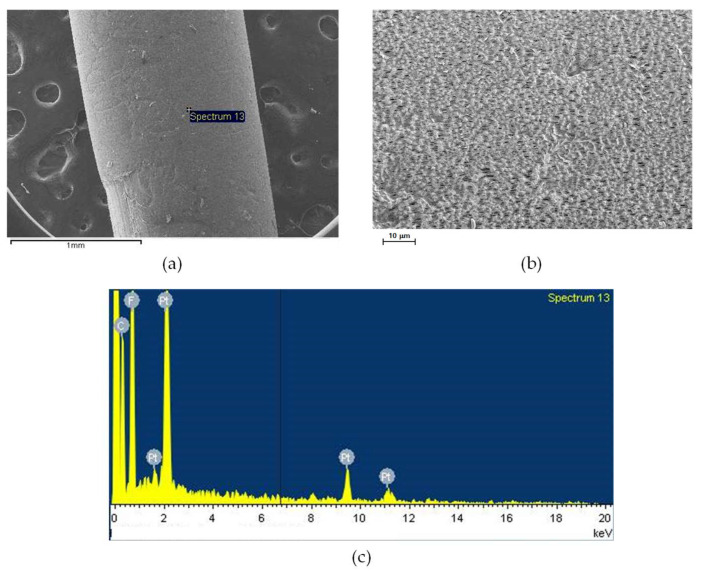
SEM images of an intact membrane fiber: (**a**) fiber surface, (**b**) surface morphology at higher magnification, and (**c**) EDS spectrum showing elemental composition.

Figure 8 presents the SEM and EDS analyses of an intact PVDF membrane fiber prior to operation. As shown in Figure 8a, the membrane exhibited a clean and smooth outer surface without any visible deposits or structural defects. The higher magnification image in Figure 8b reveals a uniform porous morphology, characteristic of a well-formed PVDF membrane structure produced by the phase inversion method. The EDS spectrum (Figure 8c) confirms that the membrane primarily consists of carbon and fluorine, which are the main elements of the PVDF polymer, with platinum peaks originating from the sputter-coating process used for SEM analysis.

**Figure 9:**
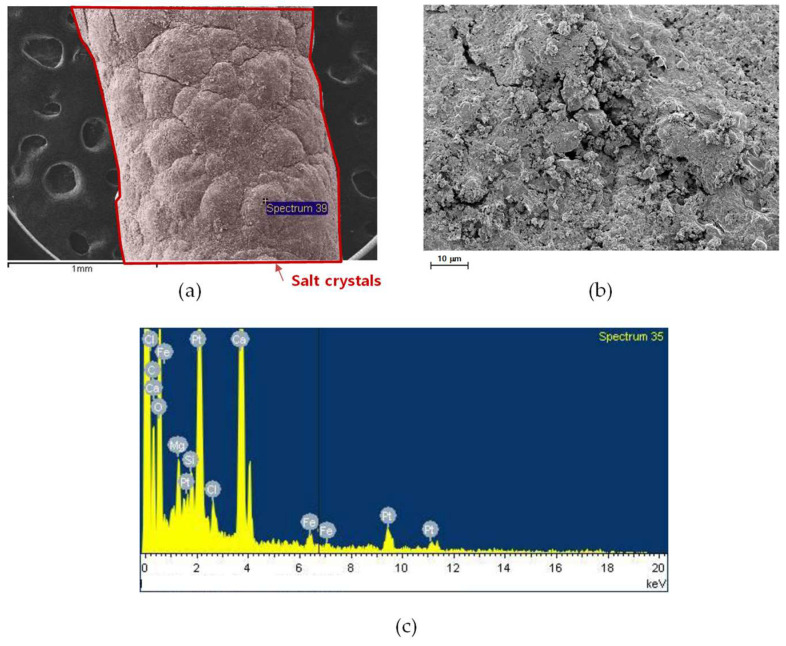
SEM images of an inner and near-inlet fiber: (**a**) fiber surface, (**b**) surface morphology at higher magnification, and (**c**) EDS spectrum showing elemental composition.

The SEM and EDS analyses of the inner and near-inlet membrane fibers after the MD operation are shown in Figure 9. A dense and uneven fouling layer was formed on the membrane surface (Figure 9a), contrasting with the smooth morphology of the intact fiber shown previously (Figure 8a). The higher magnification image in Figure 9b reveals compact and heterogeneous deposits, indicating that both particulate and inorganic fouling occurred in this region. The EDS spectrum (Figure 9c) confirms the presence of elements such as calcium (Ca), iron (Fe), and silicon (Si), suggesting that the fouling layer primarily consisted of inorganic scale and fine suspended particles originating from the feed brine. Similar results were reported in the literature: After MD operation, different forms of scales were present on the membrane surface, mainly calcium carbonate, which are commonly observed in seawater desalination operation [83]. The accumulation of such deposits near the feed inlet can be attributed to the high concentration of suspended solids in this zone, which promotes nucleation and growth of mineral scales. The literature has also reported similar results for CaCO_3_ scaling that occurred at the inlet surface of the membrane. These results demonstrate that the inner inlet region was the most susceptible to initial fouling and scaling during MD operation [83].

**Figure 10:**
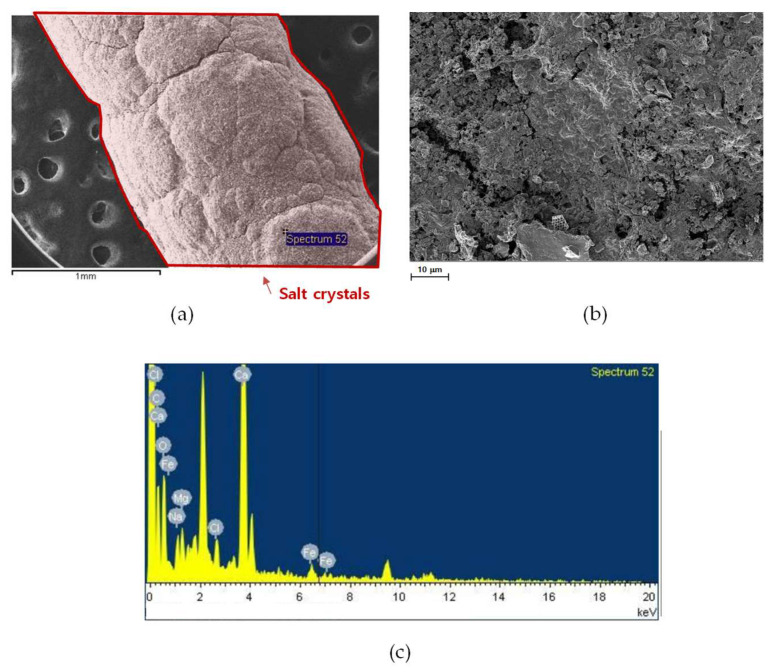
SEM images of an outer and near-inlet fiber: (**a**) fiber surface, (**b**) surface morphology at higher magnification, and (**c**) EDS spectrum showing elemental composition.

Figure 10 presents the SEM and EDS analyses of the outer and near-inlet membrane fiber after long-term operation. Compared with the inner inlet fiber shown in Figure 9, the outer fiber surface exhibited a relatively thinner and more loosely packed fouling layer (Figure 10a). The higher magnification image (Figure 10b) reveals a heterogeneous mixture of fine particles and amorphous deposits rather than the dense crystalline layer observed on the inner surface. Since the hydrodynamic condition is one of the critical factors that influence the rate and morphology of CaCO_3_ deposition [84], the inner and outer membrane fibers showed different morphologies. The EDS spectrum (Figure 10c) shows the presence of Ca, Mg, Fe, and Cl, which are similar to the EDS spectrum in Figure 9. These results suggest that fouling intensity decreases radially from the inner to the outer fiber zones within the MD module, but the foulant compositions are similar.

**Figure 11:**
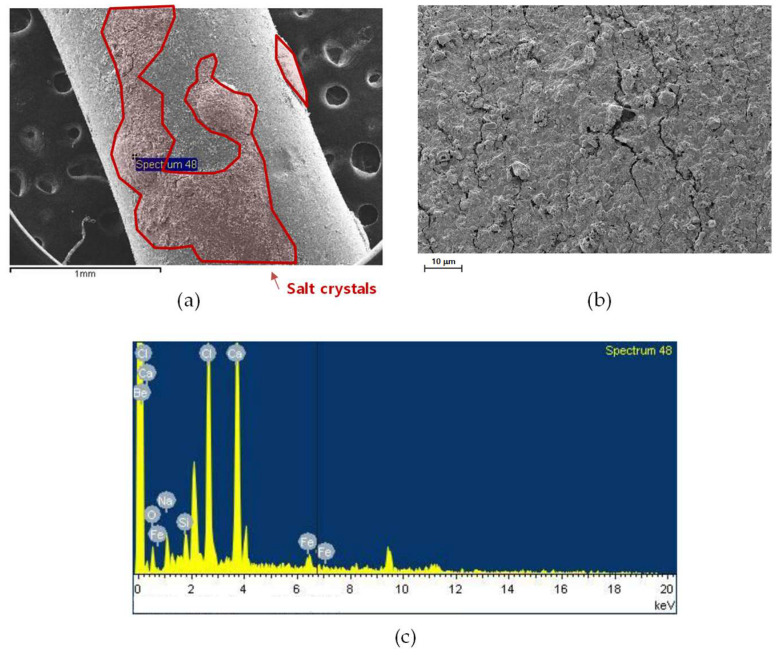
SEM images of an inner and near-outlet fiber: (**a**) fiber surface, (**b**) surface morphology at higher magnification, and (**c**) EDS spectrum showing elemental composition.

**Figure 12:**
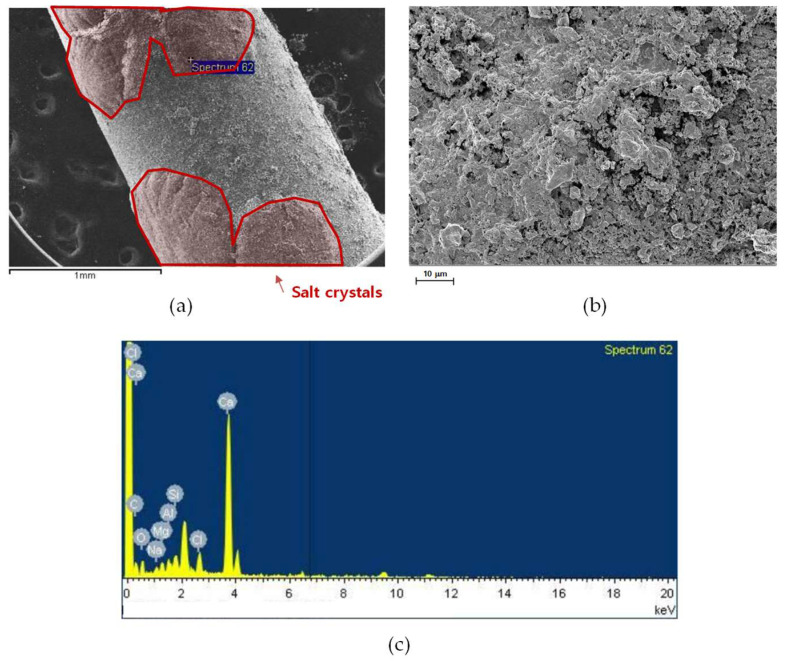
SEM images of an outer and near-outlet fiber: (**a**) fiber surface, (**b**) surface morphology at higher magnification, and (**c**) EDS spectrum showing elemental composition.

The SEM and EDS analyses of the inner and outer fibers located near the outlet of the MD module are shown in Figure 11 and Figure 12. Compared with the inlet regions (Figure 9 and Figure 10), both fibers exhibited thinner and less compact fouling layers, indicating that foulant deposition was more pronounced at the inlet. As shown in Figure 11a and Figure 12a, the membrane surfaces near the outlet were covered by relatively uniform but thinner deposits. The higher-magnification images (Figure 11b and Figure 12b) show a slightly smoother foulant layer. The EDS spectra (Figure 11c and Figure 12c) confirm the presence of Ca and O as dominant elements, accompanied by Mg, Fe, and Si, indicating that calcium carbonate was the principal inorganic foulant with minor contributions from other compounds.

**Figure 13:**
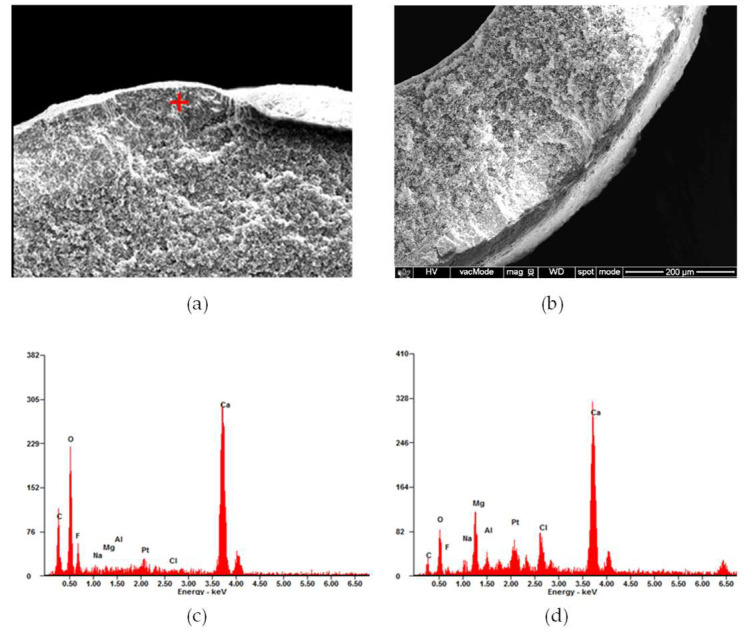
Cross-sectional SEM and EDS analyses of fouled membrane fibers: (**a**) cross-sectional view of the fouled near-inlet fiber, (**b**) cross-sectional view of the fouled near-outlet fiber, (**c**) EDS spectrum of the fiber near inlet, and (**d**) EDS spectrum of the fiber near outlet.

Cross-sectional SEM analysis was also conducted to investigate foulants within the membrane inner structure and to confirm the presence of deposits inside the pores. As shown in Figure 13a, a thick fouling layer was observed on the surface of the near-inlet fiber, indicating that fouling occurred not only on the external surface but also within the membrane matrix. In contrast, the near-outlet fiber (Figure 13b) exhibited a thinner and less compact deposit layer, suggesting reduced fouling intensity toward the outlet. The EDS spectra (Figure 13c,d) show that Ca and O were the predominant elements, confirming that the deposits mainly consisted of calcium carbonate with minor contributions from magnesium and chloride salts. These findings demonstrate that inorganic scaling extended into the membrane pores, particularly in the inlet region. Similar results have been found in the literature: Scales were observed both on the membrane surface and inside the membrane pores in the MD process [85]. Such internal fouling likely contributed to the gradual decline in vapor permeability and the onset of partial wetting observed during the MD operation, as reported in the literature [86].

#### 3.3.2. Compositional Analysis

Table 7 presents the concentrations of major ionic species identified in the foulant layer extracted from the membrane fibers, normalized by membrane surface area (mg/m^2^). No detectable ions were observed in the intact membrane, confirming that the quantified species originated from operational fouling. Among the detected ions, Na^+^ and Cl^−^ were dominant, reflecting the accumulation of residual saline components from the feed brine. Considerable amounts of Ca^2+^ and Mg^2+^ were also detected, indicating the formation of inorganic scales within the module. However, calcium-based scaling appeared to be more significant than magnesium scaling. The Ca^2+^/Na^+^ and Mg^2+^/Na^+^ ratios in the feed brine were 0.040 and 0.12, respectively (Table 1). In the foulant layer, the Mg^2+^/Na^+^ ratios (0.14 and 0.11 for the near-inlet and near-outlet fibers, respectively) were comparable to those in the feed, whereas the Ca^2+^/Na^+^ ratios (0.19 and 0.14) were 3.5–4.3 times higher. This enrichment in calcium relative to sodium suggests that Ca^2+^ preferentially precipitated as solid scale within the membrane module. This result aligns with the literature: Magnesium scaling typically occurs as magnesium hydroxide (Mg(OH)_2_) or magnesium carbonate, but is less prevalent and generally forms after significant calcium scaling has already occurred [72].

The near-outlet fiber exhibited slightly higher Na^+^ and Cl^−^ concentrations (254.7 and 501.8 mg/m^2^, respectively) than the near-inlet fiber (198.2 and 428.4 mg/m^2^), likely due to progressive salt concentration along the feed flow path. In contrast, Ca^2+^ and Mg^2+^ concentrations remained relatively uniform between the inlet and outlet regions, implying that scaling occurred throughout the module. Notably, the higher Ca^2+^/Na^+^ ratio in the near-inlet fiber (0.19) compared to the near-outlet fiber (0.14) indicates that calcium scale formation was more pronounced near the inlet, consistent with the higher foulant loading and higher temperature polarization observed in this region [87].

The total organic carbon (TOC) analysis (Table 8) revealed the presence of a measurable amount of organic matter in the foulant layer. The TOC concentration was higher in the near-inlet fibers (5.17 ± 0.13 mg/m^2^) than in the near-outlet fibers (4.07 ± 0.14 mg/m^2^), indicating that organic fouling was more pronounced at the inlet region. This result suggests that organic compounds present in the feed brine were preferentially adsorbed as the solution first contacted the membrane surface [88], forming an initial conditioning layer that promoted subsequent inorganic deposition. According to the literature, the organic compounds found in seawater and desalination brines are mainly natural organic material (NOM) such as humic and fulvic acids (about 80% of dissolved organic carbon), with the remainder consisting of carbohydrates, carboxylic acids, amino acids, and hydrocarbons [89]. Although the total organic content was relatively low compared with the concentration of inorganic species, the adsorbed organic matter likely enhanced the adhesion of mineral particles and facilitated the formation of a composite fouling layer [90], particularly in the inlet zone of the MD module.

#### 3.3.3. FT-IR Analysis

**Figure 14:**
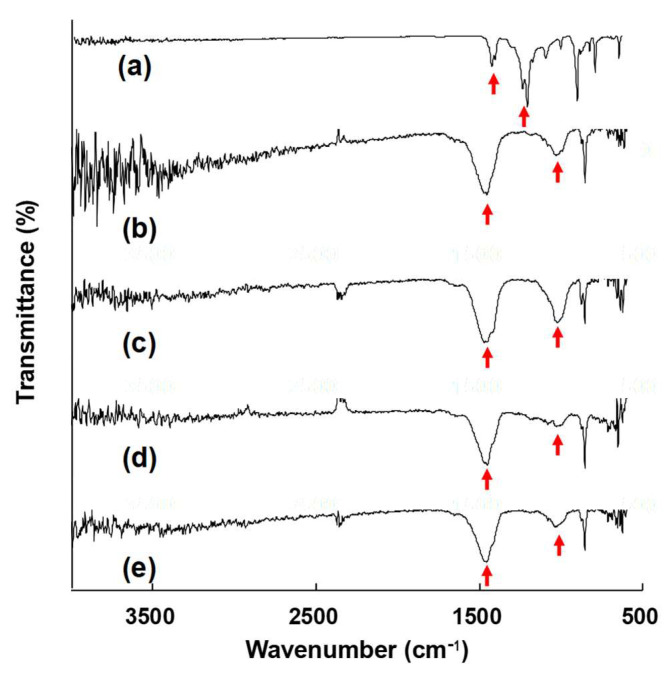
Fourier transform infrared (FT-IR) spectra of intact and fouled membranes: (**a**) intact fiber, (**b**) inner and near inlet fiber, (**c**) inner and near outlet fiber, (**d**) outer and near inlet fiber, (**e**) outer and near outlet fiber; red arrows mark the characteristic FT-IR absorption peaks selected for comparison among the membrane samples.

FT-IR spectra were collected for the pristine PVDF membrane and for fouled segments after 120 days. The pristine spectrum (Figure 14a) shows characteristic PVDF bands (C–F/CH_2_ modes) in the ~1180–1400 cm^−1^ region and PVDF skeletal features near ~840–880 cm^−1^ [91]. In contrast, all fouled specimens (Figure 14b–e) exhibit superimposed carbonate signatures: a strong ν_3_(CO_3_^2−^) band at 1410–1460 cm^−1^ and the diagnostic ν_2_(CO_3_^2−^) out-of-plane bend at 870–880 cm^−1^ [92], confirming CaCO_3_ scaling. The broad envelope at 3200–3600 cm^−1^ is attributed to ambient moisture and is not used for interpretation [93]. These results indicate CaCO_3_ is the dominant deposit, with minor hydrated/organic residues, consistent with the EDS findings.

### 3.4. Proposed Mechanism of MD Module Fouling and Wetting

**Figure 15:**
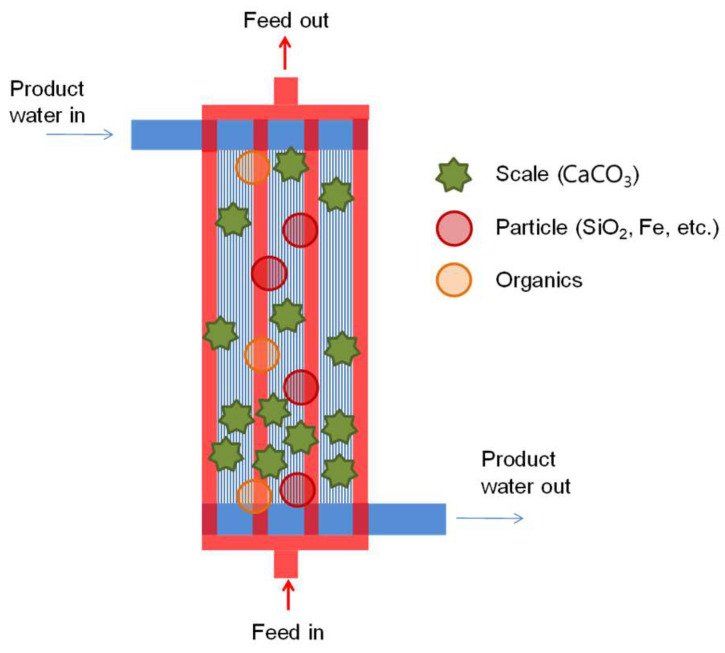
Schematic illustration of the fouling mechanism in the hollow fiber MD module. Foulants, including inorganic scale (CaCO_3_), suspended particles (SiO_2_, Fe, etc.), and organic matter, progressively accumulate within the module during operation.

Our new findings from morphological, elemental, and chemical analyses are as follows: (1) Fouling was spatially non-uniform, with the greatest severity near the inlet and inner fibers; (2) deposits reduced surface hydrophobicity and LEP, corroborating a scaling-enabled wetting pathway linked to performance loss; and (3) a larger amount of organic matter (~27% more) was deposited on the fibers near the inlet than on those near the outlet. Based on these findings, the fouling mechanism in the MD module is proposed as illustrated in Figure 15. The fouling layer primarily consisted of inorganic scales (CaCO_3_), suspended particles (SiO_2_, Fe, etc.), and organic matter, which accumulated on the membrane surface and within the pore structure during operation. Fouling was more pronounced in the inlet region due to the higher feed temperature and greater concentrations of suspended particles and organic compounds, which facilitated calcium carbonate nucleation and growth. As the feed progressed toward the outlet, the lower concentrations of particulate and organic matter, along with reduced temperature, resulted in weaker scaling tendencies and thinner deposits. Furthermore, fouling was more severe in the inner part of the module, where limited flow mixing and localized stagnation enhanced temperature and concentration polarization. These combined effects led to preferential deposition of inorganic and organic foulants in the inner and inlet zones, ultimately accelerating flux decline and partial wetting during long-term operation.

## 4. Conclusions

In this study, a pilot-scale hollow fiber membrane distillation (MD) module was operated continuously with real desalination brine to evaluate its long-term performance and fouling behavior. The following conclusions were withdrawn:

1. The DCMD module treating real MED brine maintained >99% salt rejection over 120 days, while flux and permeability declined due to progressive fouling and partial wetting.
2. Autopsy identified CaCO_3_ scaling as the dominant deposit, with minor SiO_2_/Fe particulates and trace organics; SEM/EDS, ICP-OES, and FT-IR provided consistent evidence.
3. Fouling was spatially non-uniform—most severe near the inlet and inner fibers—where higher local temperature and weaker mixing promoted supersaturation and deposition.
4. Deposits reduced surface hydrophobicity and LEP, corroborating a scaling-enabled wetting pathway linked to performance loss.
5. The results highlight the need for improved hydrodynamic design, targeted pretreatment/antiscalants, and carbonate-oriented cleaning to sustain long-term operation; future work will evaluate anti-scaling membranes, optimized flow channels, and hybrid MD configurations for robust brine management.
6. To ensure reliability and reproducibility, multiple parallel membrane modules should be tested simultaneously to minimize inaccuracies in conclusions caused by experimental deviations. Accordingly, future work will include parallel module testing to statistically validate the observed spatial fouling trends.

## Figures and Tables

**Table 1:** Summary of water quality parameters for desalination brine.

Component	Concentration (mg/L)
Ca^2+^	725.2
Mg^2+^	2156
Na^+^	18,312
K^+^	915.6
HCO_3_^−^	212.8
SO_4_^2−^	4830
Cl^−^	36,540
TDS	65,520
Total hardness (as CaCO_3_)	10,654
Total suspended solids	26.6

**Table 2:** Summary of the MD membrane module.

Parameter	Value
Materials	Polyvinylidene fluoride.
Module type	Hollow fiber (outside-in)
Flow configuration	Counter current
Inner diameter (mm)	1.2
Outer diameter (mm)	0.7
Thickness (mm)	0.25
Tensile strength (MPa)	9.05
Elongation ratio (%)	70
Effective membrane area (m^2^)	2.3

**Table 3:** Summary of operating conditions for the MD pilot plant.

Parameter	Value
Operation mode	Direct contact MD
Feed temperature	55~60 °C
Feed flow rate	60 L/min
Product water flow rate	40 L/min
Feed tank volume	0.1 m^3^
Nominal capacity	0.1 m^3^/day

**Table 4:** Comparison of contact angles for different membrane fiber samples.

Fiber Position	Contact Angle (MPa)
Intact membrane fiber	104.65
Inner and near-inlet fiber	47.05
Outer and near-inlet fiber	59.24
Inner and near-outlet fiber	41.26
Outer and near-outlet fiber	53.73

**Table 5:** Comparison of liquid entry pressures (LEPs) for different membrane fiber samples.

Fiber Position	LEP (Bar)
Intact membrane fiber	2.78 ± 0.67
Inner and near-inlet fiber	1.21 ± 0.86
Outer and near-inlet fiber	1.70 ± 0.85
Inner and near-outlet fiber	1.18 ± 0.40
Outer and near-outlet fiber	1.76 ± 0.06

**Table 6:** Comparison of tensile strengths for different membrane fiber samples.

Fiber Position	Tensile Strength (MPa)
Intact membrane fiber	9.05
Inner and near-inlet fiber	5.93
Outer and near-inlet fiber	6.36
Inner and near-outlet fiber	6.53
Outer and near-outlet fiber	6.49

**Table 7:** Ionic components in the foulant layer at the feed water inlet and outlet of the membrane module [mg/m^2^].

Position	Na^+^	Mg^2+^	Ca^2+^	K^+^	Cl^−^
Intact membrane fiber	-	-	-	-	-
Near-inlet fiber (Inner and outer)	198.2	27.7	37.3	9.0	428.4
Near-outlet fiber (Inner and outer)	254.7	27.8	35.7	11.0	501.8

**Table 8:** TOC in the foulant layer at the feed water inlet and outlet of the membrane module.

Position	TOC (Measured as mg/L)	TOC (Converted as mg/m^2^)
Intact membrane fiber	-	-
Near-inlet fiber (Inner and outer)	5.3 ± 0.2	233 ± 8
Near-outlet fiber (Inner and outer)	4.0 ± 0.2	176 ± 8

## Data Availability

The original contributions presented in this study are included in the article. Further inquiries can be directed to the corresponding author.

## Abbreviations

The following abbreviations are used in this manuscript:

MD	Membrane Distillation
DCMD	Direct Contact Membrane Distillation
VMD	Vacuum Membrane Distillation
SWRO	Seawater Reverse Osmosis
MED	Multi-effect Distillation
RO	Reverse Osmosis
NF	Nanofiltration
LEP	Liquid Entry Pressure
FT-IR	Fourier Transform Infrared Spectroscopy
SEM	Scanning Electron Microscopy
EDS	Energy Dispersive X-ray Spectroscopy
ICP-OES	Inductively Coupled Plasma Optical Emission Spectroscopy
TOC	Total Organic Carbon
TDS	Total Dissolved Solids
TSS	Total Suspended Solids
PVDF	Polyvinylidene Fluoride
ZLD	Zero Liquid Discharge
